# A multi-ancestry genome-wide association study in type 1 diabetes

**DOI:** 10.1093/hmg/ddae024

**Published:** 2024-03-07

**Authors:** Dominika A Michalek, Courtney Tern, Wei Zhou, Catherine C Robertson, Emily Farber, Paul Campolieto, Wei-Min Chen, Suna Onengut-Gumuscu, Stephen S Rich

**Affiliations:** Center for Public Health Genomics, University of Virginia, 1330 Jefferson Park Avenue, Charlottesville, VA 22908, United States; Center for Public Health Genomics, University of Virginia, 1330 Jefferson Park Avenue, Charlottesville, VA 22908, United States; Analytic and Translational Genetics Unit, Department of Medicine, Massachusetts General Hospital, 185 Cambridge Street, Boston, MA 02114, United States; Stanley Center for Psychiatric Research, Broad Institute of MIT and Harvard, 415 Main Street, Cambridge, MA 02142, United States; Program in Medical and Population Genetics, Broad Institute of Harvard and MIT, 185 Cambridge Street, Boston, MA 02114, United States; Center for Public Health Genomics, University of Virginia, 1330 Jefferson Park Avenue, Charlottesville, VA 22908, United States; Center for Public Health Genomics, University of Virginia, 1330 Jefferson Park Avenue, Charlottesville, VA 22908, United States; Center for Public Health Genomics, University of Virginia, 1330 Jefferson Park Avenue, Charlottesville, VA 22908, United States; Center for Public Health Genomics, University of Virginia, 1330 Jefferson Park Avenue, Charlottesville, VA 22908, United States; Department of Public Health Sciences, University of Virginia, 1330 Jefferson Park Avenue, Charlottesville, VA 22908, United States; Center for Public Health Genomics, University of Virginia, 1330 Jefferson Park Avenue, Charlottesville, VA 22908, United States; Department of Public Health Sciences, University of Virginia, 1330 Jefferson Park Avenue, Charlottesville, VA 22908, United States; Center for Public Health Genomics, University of Virginia, 1330 Jefferson Park Avenue, Charlottesville, VA 22908, United States; Department of Public Health Sciences, University of Virginia, 1330 Jefferson Park Avenue, Charlottesville, VA 22908, United States

**Keywords:** type 1 diabetes, human genetics, genome-wide association study, HLA

## Abstract

Type 1 diabetes (T1D) is an autoimmune disease caused by destruction of the pancreatic β-cells. Genome-wide association (GWAS) and fine mapping studies have been conducted mainly in European ancestry (EUR) populations. We performed a multi-ancestry GWAS to identify SNPs and HLA alleles associated with T1D risk and age at onset. EUR families (N = 3223), and unrelated individuals of African (AFR, N = 891) and admixed (Hispanic/Latino) ancestry (AMR, N = 308) were genotyped using the Illumina HumanCoreExome BeadArray, with imputation to the TOPMed reference panel. The Multi-Ethnic HLA reference panel was utilized to impute HLA alleles and amino acid residues. Logistic mixed models (T1D risk) and frailty models (age at onset) were used for analysis. In GWAS meta-analysis, seven loci were associated with T1D risk at genome-wide significance: *PTPN22*, *HLA-DQA1*, *IL2RA*, *RNLS*, *INS*, *IKZF4*-*RPS26*-*ERBB3*, and *SH2B3*, with four associated with T1D age at onset (*PTPN22*, *HLA-DQB1*, *INS*, and *ERBB3*). AFR and AMR meta-analysis revealed *NRP1* as associated with T1D risk and age at onset, although *NRP1* variants were not associated in EUR ancestry. In contrast, the *PTPN22* variant was significantly associated with risk only in EUR ancestry. HLA alleles and haplotypes most significantly associated with T1D risk in AFR and AMR ancestry differed from that seen in EUR ancestry; in addition, the HLA-DRB1^*^08:02-DQA1^*^04:01-DQB1^*^04:02 haplotype was ‘protective’ in AMR while HLA-DRB1^*^08:01-DQA1^*^04:01-DQB1^*^04:02 haplotype was ‘risk’ in EUR ancestry, differing only at HLA-DRB1^*^08. These results suggest that much larger sample sizes in non-EUR populations are required to capture novel loci associated with T1D risk.

## Introduction

Type 1 diabetes (T1D) is a common autoimmune disease in which the destruction of pancreatic β-cells results in the eventual inability of the body to produce insulin [1–4]. Without insulin, there is accumulation of glucose in the bloodstream and an inability for glucose to enter cells for production of energy. Progression of glucose accumulation leads to blood vessel and organ damage from dehydration, conversion of tissue to ketones for alternative energy sources, and life-threatening diabetic ketoacidosis [5–8]. Thus, external sources of insulin are necessary for survival.

There are many risk factors associated with the development of T1D, including both genetic and generally unknown environmental factors [9–11]. As a disease of autoimmunity, T1D has been characterized by three specific stages [12]: Stage 1 represents the transition from normal glucose homeostasis in an individual with variable genetic and other risk factors to production of multiple islet autoantibodies but with glucose levels in the normal range; Stage 2 includes individuals who have multiple islet autoantibodies but with glucose levels exceeding normal range (e.g. fasting plasma glucose ≥ 100 mg/dl or ≥ 5.6 mmol/l); with Stage 3 representing clinically diagnosed T1D. Each of these stages of T1D diabetes may have overlapping, as well as distinct, genetic, and non-genetic risk factors. T1D has historically been thought to be a disease of childhood (known previously as juvenile-onset diabetes mellitus) and restricted to people of European ancestry; however, T1D occurs in individuals of all ages and ancestry groups [13–17].

The genetic basis of T1D is well-established. Twin and family studies have estimated the genetic contribution to T1D in childhood as roughly 50%. Early studies focused on the HLA region [18–20] and identified the contribution of the genes, alleles and haplotypes of the human Major Histocompatibility Complex (MHC) that have large effects on risk, specifically the HLA class I genes (*-A*, *-B*, and *-C*) and the HLA class II genes (*-DRB1*, *-DQA1*, *-DQB1*, *-DPA1*, *-DPB1*) [19–22]. Subsequent studies utilized candidate gene approaches (related to the immune response) with few (typically functional) genetic variants in small numbers of cases, controls, and families. The insulin gene (*INS*) variable number of tandem repeat (VNTR) polymorphism was identified through a candidate gene study focused on *INS* due to its direct impact on insulin metabolism and implication in risk of T1D [23]. Several additional loci were discovered containing variants with large effect that were in coding or promoter regions in candidate genes, such as *PTPN22* [24] and *CTLA4* [25].

With the development of rapid genotyping technology and ability to assemble large numbers of cases with T1D and controls, genome-wide association studies (GWAS) became a common tool to detect genetic variants associated with disease and risk factors [26]. Initial GWAS studies identified loci that had statistical power for the detection of common variants (minor allele frequency (MAF) > 0.05) with large effect (OR > 1.5) [26], there was recognition that much of the genetic impact on common human disease would have much smaller effects, requiring significantly increased sample size. The Type 1 Diabetes Genetics Consortium (T1DGC) was formed to conduct genome-wide analyses in affected sib-pair families (linkage) and case-control collections (association) to individually increase sample size and to conduct meta-analyses in T1D [27]. Following GWAS, additional genotyping for fine mapping required development of a custom array (ImmunoChip) to better interrogate regions of interest across the genome [28, 29]. Currently, GWAS meta-analyses and fine mapping studies [29, 30] have identified over 100 loci associated with risk of T1D.

A major limitation in most human genetic studies has been a focus on populations of European (EUR) ancestry [31–33]. Despite increasing recognition of the benefit of including diversity in discovery, scientific equity, and reducing health disparities from applications of genetic medicine, there has yet to be a GWAS in T1D that includes large sample sizes in non-EUR ancestry populations. This absence of genetic diversity represents a major gap, particularly with the increasing global incidence and prevalence of T1D [34–36]. In this report, we conduct a multi-ancestry GWAS meta-analysis in cases, controls, and families with T1D for discovery of genetic variants, detection of novel ancestry-specific HLA variants and amino acid residues that are associated with risk of T1D as well as the age at onset of disease.

## Results

A total of 13 412 individuals were included in this study, with 6648 having T1D and 52% female. After genotype quality control, 430 930 variants were included for imputation. Each individual’s genetic ancestry was inferred by using multi-dimensional scaling (MDS), where T1DGC samples were projected to 1000 Genome phase-3 reference panel (see Methods/Population stratification for details). Each participant was assigned to one of three genetic ancestry groups—African (AFR), Admixed (AMR) and European (EUR). Genotypes were imputed using the TOPMed multi-ancestry reference panel. After additional SNP quality control, 13 777 800 (AFR), 8 952 895 (AMR), and 8 500 361 (EUR pseudo case-control) variants (MAF > 0.01) remained for association analyses. Association analyses were conducted separately on individual ancestry groups (409 AFR cases, 482 AFR controls; 153 AMR cases, 155 AMR controls; and 3428 pseudo-cases and 3428 pseudo-controls generated from affected sibpair families). Across ancestry groups, the average age at onset of T1D ranges from 8.3–11.0 years, with more females than males (67% in AFR, 58% in AMR and 51% in EUR) (Supplementary Table 1, Supplementary Fig. 1).

### Genome-wide association analysis of T1D across diverse ancestry

Logistic mixed models were fit for each ancestry group. In meta-analysis of AFR, AMR and pseudo case-control datasets, there was no evidence of systematic bias (λ_GC_ = 1.01) after excluding SNPs in the MHC region. Seven known T1D-associated loci were identified with genome-wide significance (*P* < 5.0 × 10^−8^): 1p13.2 (*PTPN22*), 6p21.32 (*HLA-DQA1*), 10p15.1 (*IL2RA*), 10q23.31 (*RNLS*), 11p15.5 (*INS*), 12q13.2 (*IKZF4-RPS26-ERBB3*), and 12q24.12 (*SH2B3*) (Table 1; Supplementary Fig. 2A and B, Supplementary Fig. 3A–F). The *INS* SNP rs689 exhibited the strongest association among non-HLA region SNPs (OR = 1.81, P = 2.34 × 10^−45^). The non-HLA region lead variant, rs6679677 in the *PTPN22* locus, is in high linkage disequilibrium (LD) (r^2^ ~ 0.96) with the known rs2476601 (R620W) SNP, residing in the coding sequence of *PTPN22* [24, 37, 38]. The risk allele frequency of rs2476601 (in perfect LD with rs6679677 in EUR populations) differs across ancestry groups. The EUR ancestry population only exhibited robust evidence of association with T1D; however, the allele had comparable effect sizes across ancestries, suggesting increased sample sizes in non-EUR populations are required to achieve statistical significance. The 12q13.2 region is complex, with multiple potential candidate genes associated with T1D including *IKZF4*, *RPS26*, and *ERBB3*. Previously, a EUR ancestry GWAS identified *ERBB3* as a putative candidate gene [27], while fine-mapping studies supported the *IKZF4*-*RPS26* region [28, 29]. In our diverse ancestry meta-analysis, rs7302200 was identified as the lead variant 12q13.2 (OR = 1.31, *P* = 7.74 × 10^−13^), residing between *RPS26* and *ERBB3*. Another T1D-associated region on chromosome 12 is the *SH2B3* locus, with rs597808 being the most significant variant (OR = 1.27, *P* = 4.82 × 10^−11^) and in high LD (r^2^ > 0.96) with previously identified variants in this region (rs653178 [28] and rs7310615 [29]).

**Table 1:** Lead variants associated with T1D risk (SAIGE).

											Heterogeneity		
Chr	BP	rsID	A1	A2	AF_A1	OR	BETA	SE	P	Direction	χ^2^	*P*-value	Gene
1	113761186	rs6679677	A	C	0.12	1.66	0.51	0.05	1.02×10^−22^	+++	0.02	0.988	*PTPN22*
2	203859027	rs1427679	A	G	0.54	0.84	−0.17	0.03	3.14×10^−7^	−	4.18	0.124	*CTLA4*
6	32619017	rs9271365	T	G	0.39	0.28	−1.29	0.03	8.80×10^−369^	−	1.76	0.414	*HLA-DQA1*
8	11734407	rs35726503	A	T	0.45	0.84	−0.18	0.03	4.00×10^−7^	−	0.24	0.885	*GATA4*
10	6052734	rs61839660	T	C	0.06	0.65	−0.43	0.08	1.79×10^−8^	?−	3.02	0.082	*IL2RA*
10	33140315	rs11009245	T	C	0.56	0.84	−0.17	0.03	5.00×10^−7^	−	13.11	0.001	*NRP1*
10	88257039	rs737391	A	G	0.18	0.78	−0.25	0.04	1.91×10^−8^	−	2.06	0.358	*RNLS*
11	2160994	rs689	A	T	0.29	0.55	−0.59	0.04	2.34×10^−45^	−	6.40	0.041	*INS*
12	56055651	rs7302200	A	G	0.32	1.31	0.27	0.04	7.74×10^−13^	+++	0.33	0.848	*IKZF4*-*RPS26*-*ERBB3*
12	111535554	rs597808	A	G	0.46	1.27	0.24	0.04	4.82×10^−11^	+++	1.34	0.512	*SH2B3*
22	30037182	rs35829240	G	GT	0.49	0.84	−0.17	0.03	4.63×10^−7^	−	0.59	0.745	*HORMAD2*

The number of cases and controls used in the analyses and the ancestry-specific results are provided in Supplementary Tables 1 and 2.

Genome Build: GRCh38.

A1—effect allele.

A2—alternative allele.

AF_A1—allele frequency of the effect allele.

OR—odds ratio.

BETA—effect size.

SE—standard Error of BETA.

P—*P*-value for meta-analysis of 3 ancestries.

Direction—indication of risk (+), protection (−) or missing SNP (?) for AFR, AMR and EUR.

χ^2^—heterogeneity statistic from Cochran’s test.

*P*-value—heterogeneity *P*-value.

Gene—the nearest or candidate gene.

Four regions reached suggestive levels of genome-wide significance (*P* < 5.0 × 10^−7^): 2q33.2 (*CTLA4*), 8p23.1 (*GATA4*), 10p11.22 (*NRP1*) and 22q12.2 (*HORMAD2*). Evidence of association for the *NRP1* locus was strongest in the AFR and AMR populations, reaching genome-wide significance (rs722988, OR_AFR_AMR_ = 1.61, P_AFR_AMR_ = 1.10 × 10^−8^) (Supplementary Table 2; Supplementary Fig. 4A) In contrast, there was little evidence of association in the EUR population (OR_EUR_ = 1.11, P_EUR_ = 0.005; P_diff_ = 0.04), suggesting potential heterogeneity (Table 1). Previously, a large fine-mapping multi-ancestry meta-analysis identified the *NRP1* locus (rs722988, OR = 1.11, *P* = 3.21 × 10^−15^) as significantly associated with T1D risk [29]. Results by ancestry can be found in Supplementary Table 2 and Supplementary Fig. 5.

### Genome-wide association analysis of T1D age at onset across diverse ancestry

AFR (N = 891), and AMR (N = 308) individuals self-reported age at onset of T1D in cases and age at enrollment for controls. In families that generated pseudo case-controls, the family members (N = 6840) self-reported their age at onset of T1D or age at enrollment. Censored time-to-event models were used for analysis of each ancestry group. There was no evidence of systematic bias (λ_GC_ = 1.01) after excluding SNPs in the MHC region. Meta-analysis of the three ancestry groups for T1D age at onset revealed four regions that attained genome-wide significance, all established T1D risk loci: 1p13.2 (*PTPN22*), 6p21.32 (*HLA-DQB1*), 11p15.5 (*INS*), and 12q13.2 (*ERBB3*) (Table 2; Supplementary Fig. 2C and D, Supplementary Fig. 3G–I). Among the non-HLA region SNPs, rs689 in the *INS* locus had the strongest association with age at onset (HR = 1.45, *P* = 2.81 × 10^−28^), like its association with T1D risk (OR = 1.81, *P* = 2.34 × 10^−45^). Although the association with T1D age at onset was weaker, the trend was consistent with disease risk. Meta-analysis revealed that rs11171747, near *ERBB3* in the 12q13.2 region, was the most significantly associated SNP with T1D age at onset (HR = 1.17, *P* = 1.20 × 10^−8^). This finding suggests that different (statistically independent) SNPs in the *IKZF4-RPS26-ERBB3* locus may be associated with T1D risk and age at onset.

**Table 2:** Lead variants associated with T1D age at onset (GATE).

											Heterogeneity		
Chr	BP	rsID	A1	A2	AF_A1	HR	BETA	SE	P	Direction	χ^2^	*P*-value	Gene
1	113761186	rs6679677	A	C	0.12	1.31	0.27	0.04	4.34×10^−11^	+++	1.06	0.588	*PTPN22*
6	32658525	rs9273364	T	G	0.50	0.39	−0.94	0.03	2.12×10^−301^	−	32.46	8.92×10^−8^	*HLA-DQB1*
10	88287593	rs2018705	T	G	0.77	1.18	0.16	0.03	1.11×10^−7^	+++	0.27	0.873	*RNLS*
11	2160994	rs689	A	T	0.30	0.69	−0.37	0.03	2.81×10^−28^	−	2.54	0.281	*INS*
12	56124624	rs11171747	T	G	0.64	1.17	0.16	0.03	1.20×10^−8^	+++	0.57	0.753	*ERBB3*
12	111270654	rs1265564	A	C	0.58	0.86	−0.15	0.03	1.38×10^−7^	−	1.46	0.482	*SH2B3*
18	12857336	rs534911	A	G	0.74	0.85	−0.16	0.03	2.95×10^−7^	−	0.17	0.917	*PTPN2*

The number of cases used in the analyses of age at onset is provided in Supplementary Table 1.

Genome Build: GRCh38.

A1—effect allele.

A2—alternative allele.

AF_A1—allele frequency of the effect allele.

HR—hazard ratio.

BETA—effect size.

SE—standard Error of BETA.

P—*P*-value for meta-analysis of 3 ancestries.

Direction—indication of risk (+), protection (−) or missing SNP (?) for AFR, AMR and EUR.

χ^2^—heterogeneity statistic from Cochran’s test.

P-value—heterogeneity *P*-value.

Gene—the nearest or candidate gene.

Two regions associated with T1D risk also reached suggestive evidence of association with T1D age at onset: 10q23.31 (*RNLS*) and 12q24.12 (*SH2B3*). The SNP in *RNLS* (rs2018705, HR = 1.18, *P* = 1.11 × 10^−7^) had similar direction of effect for T1D risk. The SNP with T1D in *SH2B3* (rs1265564, HR = 1.16, *P* = 1.38 × 10^−7^) is in moderate LD (r^2^ ~ 0.60) with rs597808, the variant identified as associated with T1D risk. The 18p11.21 (*PTPN2*) locus reached suggestive significance for association with T1D age at onset but not T1D risk in these data, although the *PTPN2* locus has been established previously as a T1D risk locus [39, 40]. In the *NRP1* locus, the same variant associated with T1D risk and with T1D age at onset only reached genome-wide significance in the meta-analysis of AFR and AMR ancestry subjects (rs722988, HR_AFR_AMR_ = 1.41, P_AFR_AMR_ = 2.41 × 10^−8^) (Supplementary Fig. 4B).

### HLA region class II gene and haplotype analysis in T1D

**Figure 1:**
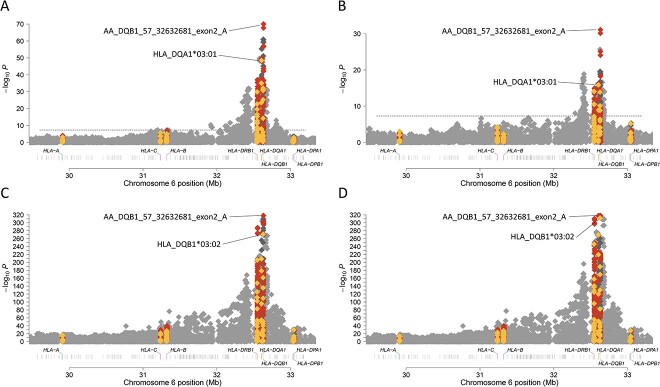
HLA alleles and amino acids associated with T1D risk in AFR (A), AMR (B), EUR (C) and meta-analysis (D). HLA class I and HLA class II genes are labeled on the x-axis. The y-axis represents −log_10_(*P*-value). The horizontal dashed line represents the threshold for genome-wide significance. SNPs are represented by grey diamonds, HLA alleles by yellow diamonds, and HLA amino acids by red diamonds. (A) T1D risk associations within the HLA region in AFR ancestry individuals. (B) T1D risk associations within the HLA region in AMR ancestry individuals. (C) T1D risk associations within the HLA region in EUR ancestry individuals. (D) T1D risk associations within the HLA region in meta-analysis of AFR, AMR and EUR ancestry individuals.

**Figure 2:**
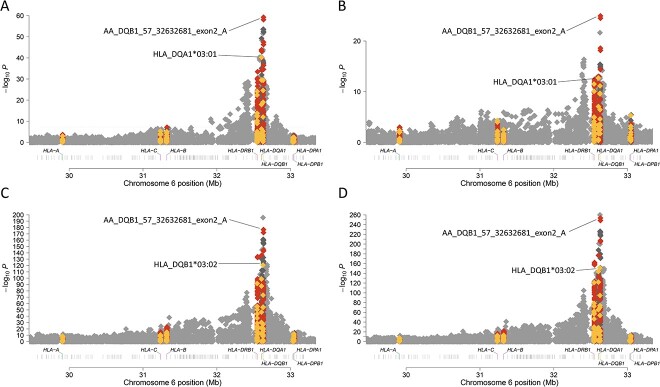
HLA alleles and amino acids associated with T1D age at onset in AFR (A), AMR (B), EUR (C) and meta-analysis (D). HLA class I and HLA class II genes are labeled on the x-axis. The y-axis represents −log_10_(*P*-value). The horizontal dashed line represents the threshold for genome-wide significance. SNPs are represented by grey diamonds, HLA alleles by yellow diamonds, and HLA amino acids by red diamonds. (A) T1D age at onset associations within the HLA region in AFR ancestry individuals. (B) T1D age at onset associations within the HLA region in AMR ancestry individuals. (C) T1D age at onset associations within the HLA region in EUR ancestry individuals. (D) T1D age at onset associations within the HLA region in meta-analysis of AFR, AMR and EUR ancestry individuals.

The association analyses of T1D with the HLA region included AFR ancestry (409 cases and 482 controls), AMR ancestry (153 cases, 155 controls), and EUR ancestry (2970 pseudo-cases, 2970 pseudo-controls). After imputation, the HLA region contained 20 329 variants for AFR, 20 376 variants for AMR, and 20 279 variants for EUR). Classical HLA alleles and HLA gene amino acid sequences were imputed from SNP data (Methods). In AFR and AMR ancestry groups, the most significantly associated allele for both T1D risk and age at onset was HLA-DQA1^*^03:01. In the EUR ancestry group, however, the HLA-DQB1^*^03:02 allele was most associated with both T1D risk and age at onset. When we evaluated amino acid residues in HLA genes, the established HLA-*DQB1* amino acid position 57 was associated most strongly with both T1D risk (Fig. 1) and age at onset (Fig. 2) across the three ancestry groups.

It has been documented that the combination (haplotypes) of specific *HLA*-*DRB1*, -*DQA1* and -*DQB1* alleles have been associated with the risk of T1D [41, 42]. To identify the ancestry-specific risk alleles of *HLADR-DQ* haplotypes, three locus HLA class II haplotypes were analyzed. The most significantly associated haplotype with T1D in AFR and AMR (Table 3) was *HLA*-DRB1^*^03:01-DQA1^*^05:01-DQB1^*^02:01 (OR_AFR_ = 4.23, P_AFR_ = 1.9 × 10^−22^; OR_AMR_ = 6.95, P_AMR_ = 2.6 × 10^−10^). In EUR ancestry (Table 3), the *HLA*-DRB1^*^04:01-DQA1^*^03:01-DQB1^*^03:02 haplotype (OR_EUR_ = 6.66, P_EUR_ = 4.5 × 10^−207^) was the most significantly associated with T1D.

**Table 3:** Association of MHC class II haplotypes with T1D in pseudo case–control European, African and admixed ancestry individuals.

HLA haplotype			EUR				AFR				AMR			
DRB1	DQA1	DQB1	control AF	case AF	OR^a^	P	control AF	case AF	OR^a^	P	control AF	case AF	OR^a^	*P*
01:01	01:01	05:01	0.097	0.064	0.63	2.3×10^−10^	0.026	0.032	1.16	6.2×10^−1^	.	.	.	.
01:02	01:01	05:01	0.014	0.008	0.54	1.1×10^−3^	0.031	0.030	0.99	9.8×10^−1^	.	.	.	.
01:03	01:01	05:01	0.009	0.004	0.44	1.2×10^−3^	.	.	.	.	.	.	.	.
03:01	05:01	02:01	0.152	0.332	3.10	5.0×10^−117^	0.069	0.245	4.23	1.9×10^−22^	0.048	0.233	6.95	2.6×10^−10^
03:02	04:01	04:02	.	.	.	.	0.056	0.009	0.17	1.1×10^−7^	.	.	.	.
04:01	03:01	03:01	0.042	0.020	0.48	7.8×10^−11^	.	.	.	.	.	.	.	.
04:01	03:01	03:02	0.062	0.261	6.66	4.5×10^−207^	0.016	0.088	6.17	6.3×10^−12^	.	.	.	.
04:02	03:01	03:02	0.017	0.042	2.65	4.5×10^−15^	.	.	.	.	0.008	0.107	12.88	8.8×10^−7^
04:03	03:01	03:02	0.006	0.003	0.51	2.1×10^−2^	.	.	.	.	.	.	.	.
04:04	03:01	03:02	0.036	0.055	1.60	5.8×10^−7^	.	.	.	.	0.072	0.088	1.31	4.1×10^−1^
04:05	03:01	02:01	0.002	0.007	3.39	1.1×10^−4^	.	.	.	.	.	.	.	.
04:05	03:01	03:02	0.007	0.026	4.21	1.1×10^−16^	0.009	0.084	9.95	1.4×10^−14^	.	.	.	.
04:07	03:01	03:01	0.008	0.001	0.06	1.1×10^−11^	.	.	.	.	.	.	.	.
04:07	03:01	03:02	.	.	.	.	.	.	.	.	0.088	0.099	1.20	5.5×10^−1^
07:01	02:01	02:01	0.100	0.039	0.37	1.9×10^−36^	0.075	0.057	0.73	1.4×10^−1^	0.076	0.046	0.50	8.4×10^−2^
07:01	02:01	03:03	0.027	0.002	0.07	8.3×10^−32^	.	.	.	.	.	.	.	.
07:01	03:01	02:01	.	.	.	.	0.008	0.049	6.68	8.2×10^−8^	.	.	.	.
08:01	04:01	04:02	0.025	0.031	1.25	5.8×10^−2^	.	.	.	.	.	.	.	.
08:02	04:01	04:02	.	.	.	.	.	.	.	.	0.120	0.031	0.25	3.4×10^−4^
08:04	04:01	03:01	.	.	.	.	0.038	0.009	0.24	1.1×10^−4^	.	.	.	.
09:01	03:01	02:01	.	.	.	.	0.037	0.102	2.99	1.0×10^−7^	.	.	.	.
09:01	03:01	03:03	0.013	0.011	0.87	4.3×10^−1^	.	.	.	.	.	.	.	.
10:01	01:01	05:01	0.008	0.002	0.22	1.2×10^−6^	.	.	.	.	.	.	.	.
11:01	05:01	03:01	0.056	0.009	0.16	4.2×10^−47^	0.032	0.011	0.32	2.1×10^−3^	.	.	.	.
11:02	05:01	03:01	.	.	.	.	0.052	0.009	0.17	3.1×10^−7^	.	.	.	.
11:04	05:01	03:01	0.029	0.003	0.08	4.0×10^−34^	.	.	.	.	.	.	.	.
12:01	01:01	05:01	.	.	.	.	0.032	0.011	0.33	3.1×10^−3^	.	.	.	.
12:01	05:01	03:01	0.013	0.004	0.32	2.2×10^−7^	.	.	.	.	.	.	.	.
13:01	01:03	06:03	0.057	0.012	0.19	3.7×10^−42^	.	.	.	.	.	.	.	.
13:02	01:02	06:04	0.036	0.026	0.72	3.0×10^−3^	0.014	0.024	1.72	1.5×10^−1^	.	.	.	.
13:02	01:02	06:09	.	.	.	.	0.019	0.017	0.89	7.5×10^−1^	.	.	.	.
13:03	05:01	03:01	0.010	0.002	0.18	3.0×10^−9^	.	.	.	.	.	.	.	.
14:01	01:01	05:03	0.013	0.001	0.04	3.0×10^−19^	.	.	.	.	.	.	.	.
15:01	01:02	06:02	0.107	0.004	0.03	6.2×10^−151^	.	.	.	.	.	.	.	.
15:02	01:03	06:01	0.008	0.001	0.11	4.8×10^−10^	.	.	.	.	.	.	.	.
15:03	01:02	06:02	.	.	.	.	0.123	0.015	0.11	1.1×10^−18^	.	.	.	.
16:01	01:02	05:02	0.020	0.013	0.64	3.5×10^−3^	.	.	.	.	.	.	.	.

The extended results for ancestry-specific HLA haplotype analyses results are provided in Supplementary Tables 3–5.

Control AF—haplotype frequency in controls.

Case AF—haplotype frequency in cases.

OR—odds ratio.

P—*P*-value.

^a^OR adjusted.

Using conditional analysis, seven AFR ancestry HLA haplotypes (Supplementary Table 3), two AMR ancestry HLA haplotypes (Supplementary Table 4), and nineteen EUR ancestry HLA haplotypes (Supplementary Table 5) were independently associated with T1D. Conditional analysis revealed that *HLA*-DRB1^*^08:02-DQA1^*^04:01-DQB1^*^04:02 haplotype was protective (OR < 1) in the AMR ancestry group (OR = 0.39, *P* = 2.9 × 10^−2^). In the EUR ancestry group, the *HLA*-DRB1^*^08:01-DQA1^*^04:01-DQB1^*^04:02 haplotype (differing only at the second field of *HLA*-DRB1^*^08) was associated with increased risk for T1D (OR = 1.81, *P* = 5.5 × 10^−5^).

### Enrichment of T1D genes across autoimmune diseases

**Figure 3:**
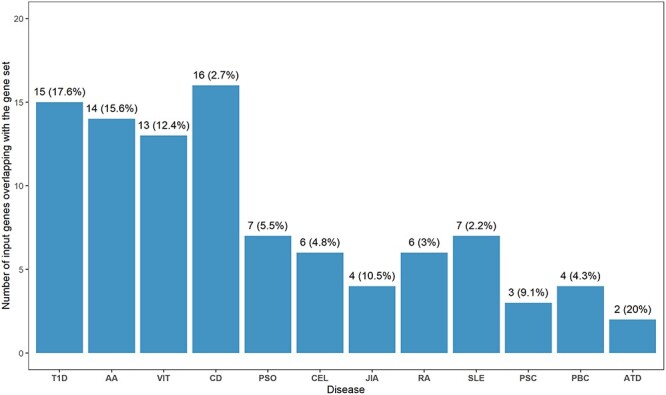
Enrichment of T1D identified genes in other autoimmune diseases. Each bar represents the number of input genes overlapping with the gene set with percentage. Diseases on the x-axis are ordered by increasing *P*-values. All presented results are significant (adj. *P*-value < 0.05). *P*-values were adjusted using a multiple test correction (Benjamini-Hochberg): T1D—4.69 × 10^−18^, AA—3.62 × 10^−16^, VIT—6.56 × 10^−14^, CD—1.54 × 10^−7^, PSO—5.96 × 10^−5^, CEL—5.79 × 10^−4^, JIA—8.23 × 10^−4^, RA—5.81 × 10^−3^, SLE—8.79 × 10^−3^, PSC—1.03 × 10^−2^, PBC—1.50 × 10^−2^, ATD—1.87 × 10^−2^. The MHC region (chr6: 25 Mb-35 Mb) was excluded from the analysis. AA—Alopecia Areata, ATD—Autoimmune Thyroid Disease, CEL—Celiac Disease, CD—Crohn’s Disease, JIA—Juvenile Idiopathic Arthritis, PBC—Primary Biliary Cholangitis, PSC—Primary Sclerosing Cholangitis, PSO—Psoriasis (PSO), RA—Rheumatoid Arthritis, SLE—Systemic Lupus Erythematosus, T1D—Type 1 Diabetes, VIT—Vitiligo.

The coexistence of T1D with other immune-mediated diseases was documented extensively through clustering in families in part due to similarity of HLA associations with disease. Recent evidence of genetic similarity of autoimmune diseases has been shown in analysis of targeted array data and not genome-wide analysis. We utilized GWAS data to compare enrichment of T1D-annotated genes against GWAS-catalog reported genes in autoimmune diseases (Fig. 3) using FUMA software [43]. We identified 12 autoimmune diseases, including T1D that shared annotated genes. The most significant overlap of identified T1D genes in autoimmune diseases was with alopecia areata (AA; *P* = 3.62 × 10^−16^). The overlap between T1D and AA loci was driven, in part, by common susceptibility variants within *PTPN22*, *IL2RA*, *IKZF4*, *ERBB3* and *SH2B3*. In EUR ancestry populations, previous gene set enrichment analysis (using the fine-mapping ImmunoChip array) with T1D showed a similar overlap with diseases that have characteristic tissue autoantibodies; e.g. AA, juvenile idiopathic arthritis (JIA), and rheumatoid arthritis (RA) [28].

## Discussion

This is the first genome-wide association scan of T1D in populations of diverse ancestry. While previous studies utilized targeted genotyping arrays (ImmunoChip), we demonstrated that no additional loci could be identified genome-wide in EUR and other ancestry populations. Although, the study contains the largest sample sizes in AFR and AMR populations to date, much larger sample sizes will be required to fully characterize the genome with respect to T1D risk.

We identified seven regions at genome-wide significance associated with risk and four regions at genome-wide significance associated with T1D age at onset, with some regions exhibiting ancestry-specific effects. In the HLA region, we determined specific HLA class I and class II alleles and amino acids associated with T1D risk and age at onset within and across ancestry, as well as ancestry-specific HLA haplotypes. We identified seven associated HLA haplotypes in the AFR ancestry group, two in the AMR (Hispanic/Latino) ancestry group, and nineteen HLA haplotypes significantly associated with risk in the EUR ancestry group.

As expected, the strongest non-HLA regions associated with T1D risk and age at onset were seen at the known *INS* and *PTPN22* loci. The most associated variant (rs2476601, C1858T, R620W, in complete LD with rs6679677) in the *PTPN22* locus was only supported by EUR ancestry. The association between *PTPN22* rs2476601 and T1D was first documented in a case-control study of non-Hispanic white individuals from North America and Sardinia [24]. Subsequent family and case–control studies in numerous European populations [37, 44–47] confirmed its association with T1D in this locus, showing high frequencies of rs2476601 in northern European populations and decreased frequencies in southern European and Sardinian populations [48]. The *PTPN22* rs2476601 variant has been shown to be rare in African and Asian populations [48–50], supporting our results of reduced association in non-EUR ancestry individuals. In addition, it has been shown that the *PTPN22* rs2476601 SNP is associated with earlier age at onset of T1D in the European ancestry population [51].

We identified an association of the *NRP1* locus with T1D risk and age at onset in non-EUR ancestry (AFR and AMR groups) but not in the EUR ancestry group. This locus has been identified in a large fine-mapping study involving diverse ancestry individuals [29] and GWAS of a large European ancestry cohort [30]. Together, the *NRP1* locus may represent a region more associated with T1D risk in non-European ancestry populations, as the effect sizes are stronger in our AFR and AMR groups; however, the direction of effect is the same in all ancestry groups. In addition, the strongest associated variant in the *NRP1* locus was associated with earlier T1D age at onset. Previously, two SNPs located in intron 9 of *NRP1* were shown to be associated with T1D [52], with the strength of association stronger in children with onset before age 10 years and/or in children who had a parent with T1D. In addition, an *NRP1* isoform in pancreatic islets has been shown to be associated with a very young age at onset of T1D [53]. Cells containing the truncated version of neuropilin-1 protein (encoded by *NRP1*) are devoid of insulin, resulting in the development of T1D at a very early age. In those individuals with onset of T1D before age 4 years, the frequency of the minor allele (T) of the *NRP1* intron 9 variant (rs2070303) is increased when compared with those having an older age at onset.

Due to the importance of the HLA region in T1D and limited data in non-European ancestries, we determined ancestry-specific haplotypes in collected cohorts. We identified the *HLA*-DRB1^*^03:01-DQA1^*^05:01-DQB1^*^02:01 haplotype as the most significantly associated with T1D in non-EUR ancestry individuals (AFR and AMR). A fine-mapping study of 3949 African ancestry samples revealed the same haplotype as the strongest association [50]. In our EUR ancestry subjects, the haplotype most significantly associated with T1D was *HLA*-DRB1^*^04:01-DQA1^*^03:01-DQB1^*^03:02, consistent with previous results [54]. Comparisons across ancestries implicated differences in risk based upon *HLA*-*DRB1* gene, such as *HLA*-DRB1^*^08:02-DQA1^*^04:01-DQB1^*^04:02 as a protective haplotype in our AMR ancestry population, with *HLA*-DRB1^*^08:01-DQA1^*^04:01-DQB1^*^04:02 representing a susceptible haplotype in our EUR ancestry population.

This study has several strengths, including use of under-represented populations (African and admixed ancestry), the genome-wide coverage of variants, and imputation to increase SNP density. In addition, the detailed interrogation of the HLA region provides novel information on risk associations not only of HLA alleles but also amino acid residues and HLA haplotypes. There are, however, some limitations of the study, including the small number of samples compared with other genomic studies in European ancestry (despite having the largest non-EUR ancestry T1D genome-wide data to date). The relatively small number of samples in total may explain the number of T1D-associated loci and failure to identify new regions that would likely have small effect sizes. Despite these limitations, it is important to recognize potential ancestry-specific effects on the risk of T1D, thereby better defining the genetic landscape of T1D risk in non-European ancestry populations, particularly in the HLA region as it represents a major risk locus for T1D.

Our multi-ancestry GWAS analysis enabled the discovery of genetic variants, novel ancestry-specific HLA variants and amino acid residues associated with risk of T1D and age at T1D onset. Including subjects with diverse genetic ancestry revealed that the *NRP1* region exhibits a stronger effect on T1D risk and age at onset in non-European ancestry populations. The association of *NRP1* locus and specific HLA haplotypes, in addition to *PTPN22*, differentiate the risk of T1D between individuals of European and non-European ancestries. These results suggest that further increasing sample diversity can provide better understanding of genetic risk factors contributing to T1D in global populations. This genetic diversity could help improve population-specific genetic risk scores application to identify individuals at high genetic risk of T1D and provide opportunities for islet autoantibody screen for eligibility into early intervention trials (e.g. teplizumab) to delay or prevent disease onset.

## Materials and Methods

### Participants

We obtained DNA samples from 13 412 subjects recruited by the T1DGC (Supplementary Table 1). The study population consisted of 12 213 individuals from affected sib-pair and trio families. The majority of individuals from families were of European (EUR) ancestry (10501). The case-control series consisted of 891 unrelated individuals of AFR ancestry (409 T1D cases, 482 controls) and 308 individuals of AMR ancestry (153 T1D cases, 155 controls).

### Genotyping and quality control

All samples were genotyped according to the manufacturer’s protocol using the Illumina Infinium CoreExome BeadChip in the Genome Sciences Laboratory at the University of Virginia. Raw genotyped data were subjected to SNP-level and sample-level quality control (Supplementary Fig. 1) using KING software (version 2.2.8 [55]). For sample level quality control, we utilized chromosome X heterozygosity and chromosome Y absence to identify DNA samples that had discordant results between genetic sex and self-reported sex, these samples were treated as errors in processing and were removed. Additionally, we removed samples with a genotype call rate < 95% and evidence of Mendelian inconsistences (MI, for families). Pedigree sample relationships were updated using KING software [55]. From samples passing initial quality control metrics, the following filters were applied for variants: removal of monomorphic SNPs, removal of SNPs with call rates < 95%, removal of SNPs with Mendelian inconsistencies in > 1% of the parent-offspring pairs and trios, and removal of SNPs significantly deviating from Hardy-Weinberg Equilibrium (*P* < 1.0 × 10^−6^ [*P* < 1.0 × 10^−20^ for MHC region]). SNPs with MAF < 0.01 were not included in the analysis. Variant quality control included removal of SNPs that were not mapped uniquely to the genome (e.g. exm244817). Additional sample quality control included removal of family members with T1D or case samples with age at onset of zero, and removal of family samples with onset of diabetes greater than 32 years and with at least one affected parent (suggestive of misdiagnosis of T1D with maturity-onset diabetes of the young, MODY). All DNA samples were collected after approval from relevant institutional research ethics committees and appropriate informed consent was obtained from all subjects and families.

### Generation of a pseudo case–control sample

In the T1DGC, the family collection was ascertained specifically to include affected sib-pairs [56] followed by the collection of unrelated individuals with T1D and controls. As the analytic method (logistic mixed models) is not robust to the targeted ascertainment of affected sib-pair families, a series of pseudo-cases and pseudo-controls was generated [57] for application of the logistic mixed and frailty models (SAIGE and GATE). Within each family and for each SNP, the alleles transmitted from each parent to an affected child constituted the “pseudo case”; similarly, alleles not transmitted from each parent to an affected child constituted the “pseudo control”. From EUR affected sib-pair families, 3428 pseudo-cases and 3428 pseudo-controls were constructed; summary statistics from the pseudo case-control analysis was used in meta-analysis with the AFR and AMR case–control results.

### Population stratification

To infer genetic ancestry, we used multi-dimensional scaling (MDS) analysis implemented in KING [55]. KING utilizes a support vector machine (SVM) approach to assign an ancestry label (AFR, AMR, EUR) to each individual sample by leveraging known ancestry in the 1000 Genomes Project reference panel (https://github.com/chenlab-uva/AncestryInference_KING). Ancestry-specific principal components (PCs) were generated with principal component analysis (PCA) in control individuals, using SNPs selected by excluding the MHC region, removing SNPs with MAF ≤ 0.05, and pruning for linkage disequilibrium (r^2^ > 0.5 in 50-kb windows). Genotypes of cases were projected onto control samples using PLINK v1.9 [58] to ‘match’ for ancestry and minimize stratification effects in case–control analyses. A total of six subjects were removed that represented ‘outliers’ from the PCA projection of T1D cases onto controls.

### Imputation

Genotypes from the Illumina CoreExome BeadChip were imputed to the Trans-Omics for Precision Medicine (TOPMed) reference panel [59] on the TOPMed Imputation Server housed on the NHLBI BioData Catalyst server (https://imputation.biodatacatalyst.nhlbi.nih.gov/). For the GWAS data, a minimac4 imputation accuracy of r^2^ > 0.3 was used as a variant filter for common and infrequent variants (MAF > 0.01), with r^2^ > 0.5 used as a filter for rare variants (MAF ≤ 0.01). For both common and rare variants, all SNPs were removed with Mendelian inconsistencies (MI) in at least 10% of trio families or parent-offspring (PO) pairs. All coordinates are reported in GRCh38.

HLA imputation was conducted using the multi-ancestry HLA reference panel (HLA-TAPAS) [60] at the University of Michigan (https://imputationserver.sph.umich.edu/index.html). SNPs in the HLA region (28Mbp—34Mbp) were used to impute HLA alleles (to four-digit accuracy) and amino acid residues. The HLA-TAPAS reference panel was generated using whole genome sequencing data from ~20 000 samples from five global populations [60]. Classical alleles for HLA class I (HLA-*A*, -*B*, and -*C*) and HLA class II (HLA-*DQA1*, -*DQB1*, -*DRB1*, -*DPA1*, and -*DPB1*) genes were inferred from reads extracted from the extended MHC region by applying a population reference graph for the MHC region. Imputation accuracy was assessed by comparing the “gold standard” sequence-based typing in individuals from the 1000 Genomes Project and the Japanese cohort to inferred HLA classical alleles. For the HLA imputation, variants with r^2^ > 0.5 and MAF > 0.005 were retained. Variants were removed with MI > 10% in trio families or in parent-offspring pairs.

### Statistical analyses

Analysis of GWAS data for T1D risk (binary trait) was conducted using SAIGE software that implemented a logistic mixed model (LMM) regression approach to control for type 1 error by accounting for unbalanced case–control ratio and sample relatedness. For T1D age at onset, GATE software was used for GWAS based on a frailty model under similar unbalanced conditions.

#### Type 1 diabetes (T1D) risk (SAIGE)

GWAS data passing quality control filters (MAF > 0.01 and minor allele count (MAC) ≥ 20) were analyzed for association with T1D within three groups (pseudo case-control with majority of EUR ancestry [N = 6856], AFR [N = 891], and AMR [N = 308]). Logistic mixed model (LMM) regression implemented in SAIGE software [61] was used for analysis, which accounts for sample relatedness, adjusting for four principal components in AFR and AMR case–control groups and seven principal components in the pseudo case-control group. SAIGE controls for type I error rates even for unbalanced case–control ratios by incorporating a saddlepoint approximation (SPA) to improve estimation of the test statistic distribution at the extremes. Results of single-variant association tests were combined using a fixed-effects meta-analysis using METAL software [62]. In addition, we performed Cochran’s Q-test for heterogeneity as implemented in METAL. Association results for selected loci were plotted using LocusZoom [63, 64]. To determine the effect of population stratification in association analysis, the genomic inflation factor (λ_GC_) was estimated, defined by the ratio of median of the empirically observed distribution of the test statistic to the expected median. We performed conditional analysis on genome-wide significant (or suggestive) variants to identify loci with more than one variant independently associated with T1D risk by including the most associated SNP in the LMM and identifying the subsequent (statistically significant) SNP, adding that SNP to the model until significance was no longer present.

#### Type 1 diabetes (T1D) age at onset (GATE)

Association of GWAS variants passing filters (MAF > 0.01 and MAC ≥ 20) was evaluated with age at onset of T1D by analysis of three groups (pseudo-case pseudo-control with majority of EUR ancestry, AFR, and AMR). We used the frailty mixed model regression implemented in GATE software [65], adjusting for four principal components in AFR and AMR case–control groups and seven principal components in the pseudo case-control group. The age for all individuals with and without T1D was censored at 32 years old. Results of single-variant association tests were combined using a fixed-effects meta-analysis with METAL software.

#### HLA analysis

Association analyses of T1D risk with imputed HLA alleles and amino acid residues (MAF > 0.005 and MAC ≥ 10) were conducted in 3 ancestry-specific groups (EUR [N = 5940], AFR [N = 891], AMR [N = 381]) using SAIGE software. Association analyses of T1D age at onset using GATE software employed the same approaches. Due to missing age information, fewer samples were used for frailty mixed model analysis in EUR ancestry (N = 5930). All HLA region association analyses were adjusted for four principal components in all three ancestry groups.

To analyze association of HLA class II haplotypes with T1D, all *HLA*-*DRB1-DQA1-DQB1* haplotypes were identified by creating a matrix of all possible combinations of alleles for each sample in each ancestry-specific group. For any given locus, individuals were excluded from the analysis if the total allele count for all possible alleles at that locus did not equal two. Common HLA alleles were defined as those that occur more than 30 times in a combined group of case and control subjects. For each ancestry group (EUR, AFR, AMR), association analyses of T1D with HLA class II haplotypes (*DRB1-DQA1-DQB1*) were conducted on common haplotypes using logistic regression model and adjusting for four principal components as described previously [50]. Independent associations of *HLA*-*DRB1-DQA1-DRB1* haplotypes were assessed by conditioning on the most significant haplotype. The process was repeated until the consecutive haplotype failed to meet the significance threshold. For each ancestry group, the statistical significance was corrected for the total number of common haplotypes.

### Functional impact of detected variants

GWAS summary statistics were applied to the SNP2GENE and GENE2FUNCTION modules in FUMA v1.5.3 (Functional Mapping and Annotation, https://fuma.ctglab.nl) [43]. The SNP2GENE module was used to functionally annotate leading SNPs and the GENE2FUNCTION module was used to annotate genes and to identify genes that were enriched in pre-defined gene sets (e.g. reported genes from the GWAS catalog). Protein-coding genes were used for both foreground (T1D associated) and 20 260 background genes (for permutation analysis). We focused on enrichment of genes in the autoimmune diseases from the GWAS catalog gene set. Identified autoimmune diseases from GWAS catalog gene set included: Alopecia Areata (AA), Autoimmune thyroid diseases (ATD), Celiac disease (CEL), Crohn’s disease (CD), Juvenile Idiopathic Arthritis (JIA), Primary Biliary cholangitis (PBC), Primary Sclerosing Cholangitis (PSC), Psoriasis (PSO), Rheumatoid Arthritis (RA), Systemic Lupus Erythematosus (SLE), Type 1 Diabetes (T1D) and Vitiligo (VIT). Enrichment p-values were adjusted using a multiple test correction (Benjamini-Hochberg).

All statistical analyses and data visualization were performed using R version 4.1.1, unless otherwise stated. The HLA-TAPAS, Locus Zoom (http://locuszoom.org/) and R packages ggplot2, qqman and RColorBrewer were used for data visualization. For all conducted analyses genome-wide significance was based upon *P* < 5.0 × 10^−8^, while *P* < 5.0 × 10^−7^ was considered as suggestive evidence of association. The LD estimates were obtained from the LD pair tool (https://ldlink.nih.gov/) [66] with African (AFR), admixed (AMR) and European (EUR) cohorts’ selection.

## Supplementary Material

HMG-2023-CE-00676_ddae024_Suplementary_Tables_ddae024

HMG-2023-CE-00676_ddae024_Supplementary_Figures_ddae024

## Data Availability

Summary statistics are available in the NIH database for Genotype and Phenotype (dbGaP, https://dbgap.ncbi.nlm.nih.gov/aa/wga.cgi?page=login), with accession number phs003539.v1, and the Accelerating Medicines Partnership Common Metabolic Diseases (AMP CMD) Knowledge Portal (https://hugeamp.org/).

## Code availability

Code used to generate results is available at https://github.com/damichalek/T1DGC_GWAS.
