# Supplementary material for: A multi-ancestry genome-wide association study in type 1 diabetes
Source: Hum Mol Genet. 2024 Mar 7;33(11):958–68. doi: 10.1093/hmg/ddae024 (PMC11102596; doi:10.1093/hmg/ddae024)
Supplement: HMG-2023-CE-00676_ddae024_Supplementary_Figures_ddae024 [file hmg-2023-ce-00676_ddae024_supplementary_figures_ddae024.docx]

**Supplementary Figures**


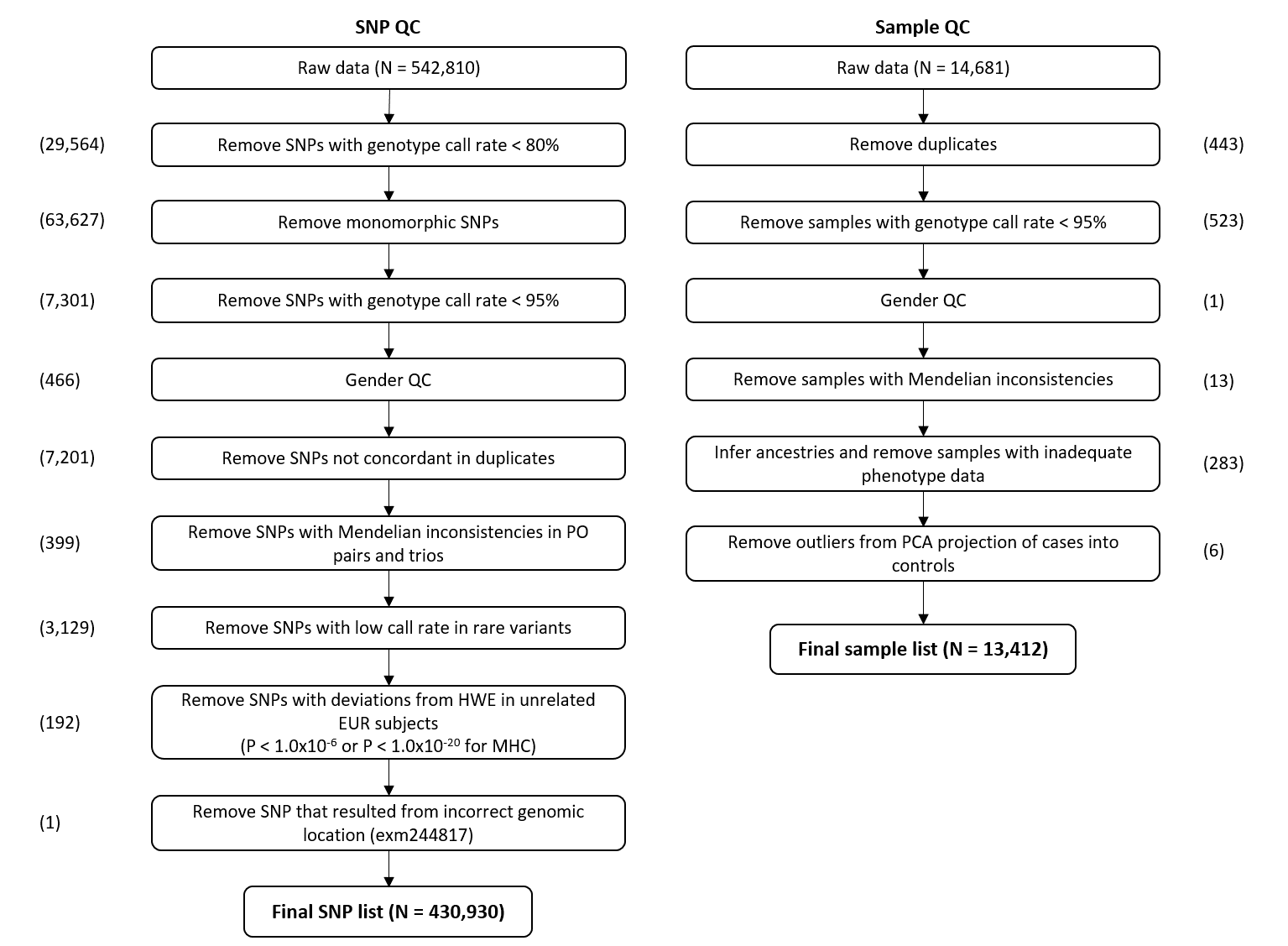


**Supplementary Figure 1.** SNP and sample quality control workflow before imputation.





**Supplementary Figure 2.** Loci associated with T1D risk (**A, B**) and age at onset (**C, D**) in AFR, AMR and pseudo case-control meta-analysis. Each locus is labeled with the nearest gene. The y-axis represents –log(P values). The horizontal red line represents the threshold for genome-wide associations. (**A**) The strongest associations with T1D risk (*HLA-DQA1*, *INS* and *PTPN22*). (**B**) Associations with T1D risk after excluding HLA region (25 Mb – 35 Mb). (**C**) The strongest associations with age at onset of T1D (*HLA-DQA1*, *INS* and *PTPN22*). (**D**) Associations with age at onset of T1D after excluding HLA region (25 Mb – 35 Mb).





**Supplementary Figure 3.** LocusZoom plots of genome-wide significant loci associated with T1D risk (**A-F**) and age at onset (**G-I**), excluding HLA region. The left y-axis represents –log(P values) and right y-axis determines recombination rate. The horizontal gray dashed line represents the threshold for genome-wide associations. The most significantly associated SNP is shown as purple diamond. For *PTPN22* locus (**A, G**), the purple diamond represents known coding region variant (rs2476601).


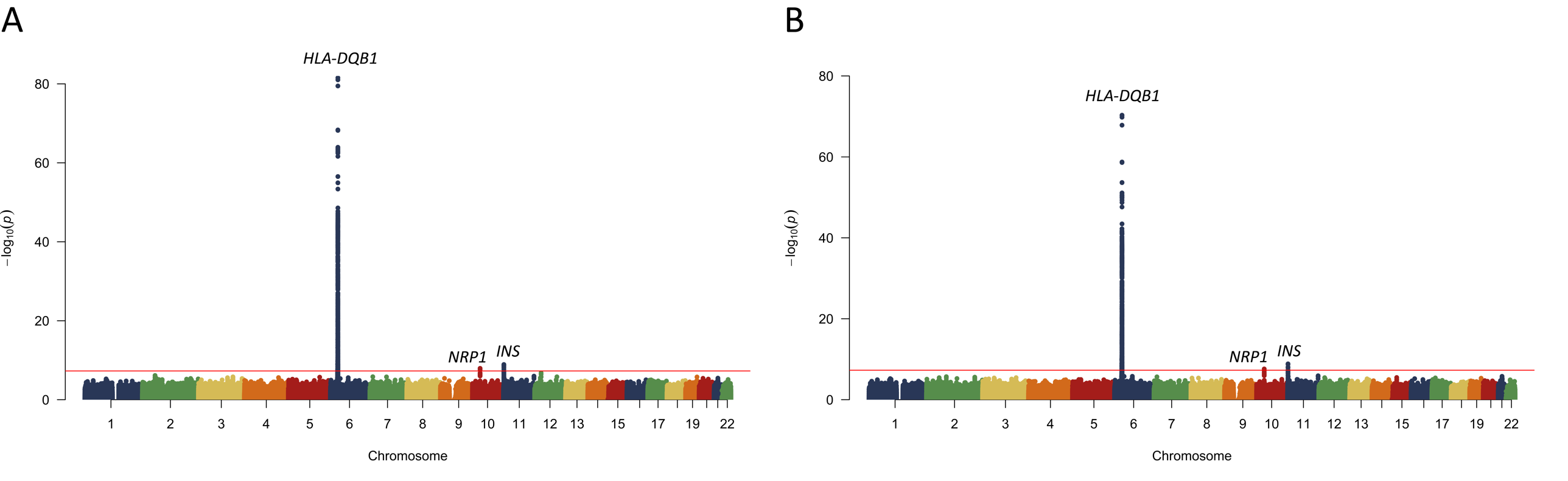


**Supplementary Figure 4.** Loci associated with T1D risk (**A**) and age at onset (**B**) in non-EUR meta-analysis (AFR and AMR). Each locus is labeled with the nearest gene. The y-axis represents –log(P values). The horizontal red line represents the threshold for genome-wide associations. (**A**) Associations with T1D risk in non-EUR meta-analysis. (**B**) Associations with age at onset of T1D in non-EUR meta-analysis.


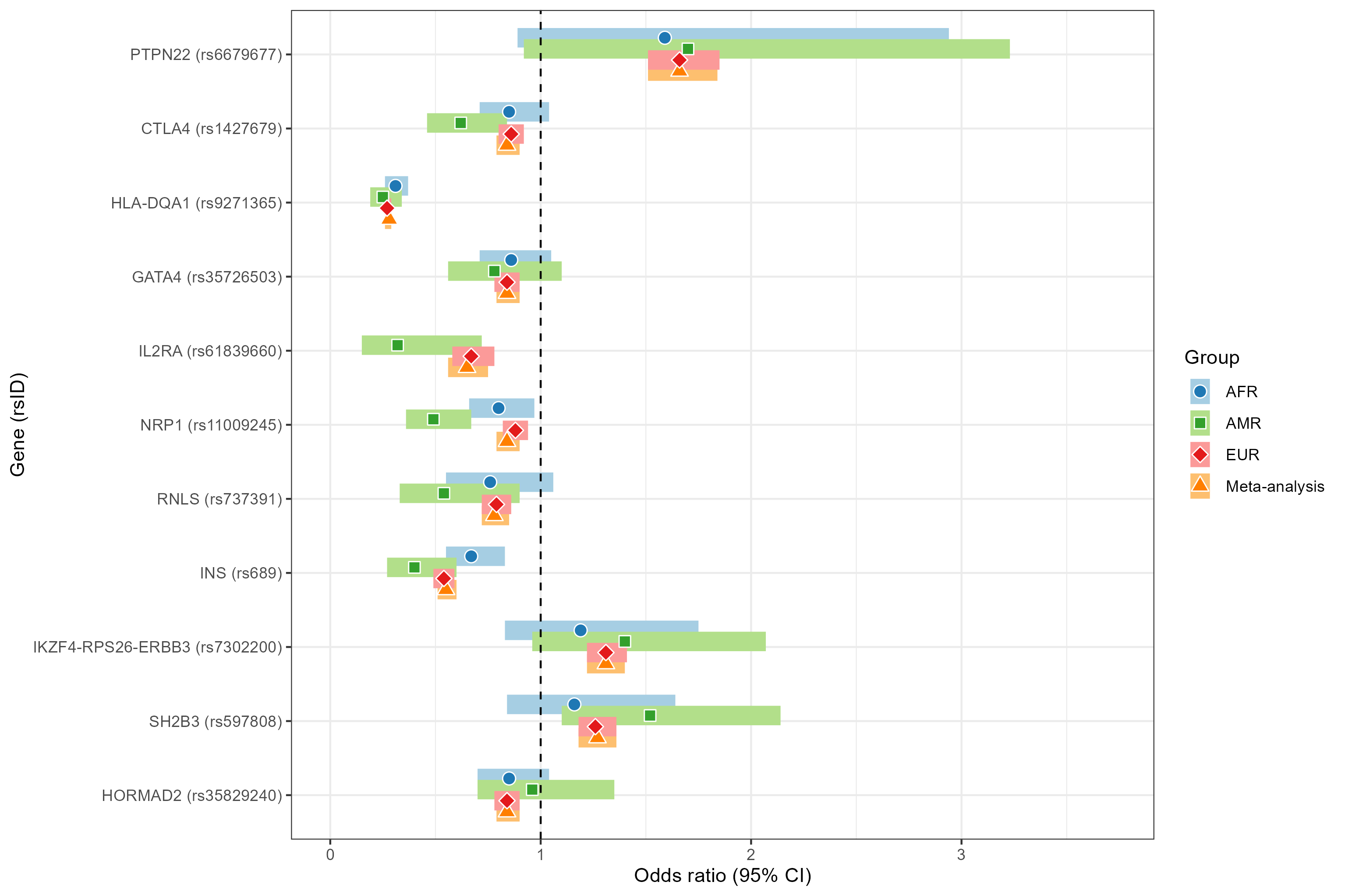


**Supplementary Figure 5.** Ancestry-specific odds ratios for T1D risk loci. The x-axis represents odds ratio with 95% CI (Confidence Interval) and y-axis shows the nearest or candidate gene. CI was calculated as [exp(BETA – 1.96SE), exp(BETA + 1.96SE)], where BETA – effect size and SE – standard error.
